# Diverse Biosynthetic Pathways and Protective Functions against Environmental Stress of Antioxidants in Microalgae

**DOI:** 10.3390/plants10061250

**Published:** 2021-06-19

**Authors:** Shun Tamaki, Keiichi Mochida, Kengo Suzuki

**Affiliations:** 1 Microalgae Production Control Technology Laboratory, RIKEN Baton Zone Program, Yokohama 230-0045, Japan; keiichi.mochida@riken.jp (K.M.); suzuki@euglena.jp (K.S.); 2 RIKEN Center for Sustainable Resource Science, Yokohama 230-0045, Japan; 3 Kihara Institute for Biological Research, Yokohama City University, Yokohama 230-0045, Japan; 4 School of Information and Data Sciences, Nagasaki University, Nagasaki 852-8521, Japan; 5 euglena Co., Ltd., Tokyo 108-0014, Japan

**Keywords:** microalgae, diversity, antioxidant, carotenoid, ascorbate, glutathione, reactive oxygen species, environmental stress

## Abstract

Eukaryotic microalgae have been classified into several biological divisions and have evolutionarily acquired diverse morphologies, metabolisms, and life cycles. They are naturally exposed to environmental stresses that cause oxidative damage due to reactive oxygen species accumulation. To cope with environmental stresses, microalgae contain various antioxidants, including carotenoids, ascorbate (AsA), and glutathione (GSH). Carotenoids are hydrophobic pigments required for light harvesting, photoprotection, and phototaxis. AsA constitutes the AsA-GSH cycle together with GSH and is responsible for photooxidative stress defense. GSH contributes not only to ROS scavenging, but also to heavy metal detoxification and thiol-based redox regulation. The evolutionary diversity of microalgae influences the composition and biosynthetic pathways of these antioxidants. For example, α-carotene and its derivatives are specific to Chlorophyta, whereas diadinoxanthin and fucoxanthin are found in Heterokontophyta, Haptophyta, and Dinophyta. It has been suggested that AsA is biosynthesized via the plant pathway in Chlorophyta and Rhodophyta and via the *Euglena* pathway in Euglenophyta, Heterokontophyta, and Haptophyta. The GSH biosynthetic pathway is conserved in all biological kingdoms; however, Euglenophyta are able to synthesize an additional thiol antioxidant, trypanothione, using GSH as the substrate. In the present study, we reviewed and discussed the diversity of microalgal antioxidants, including recent findings.

## 1. Introduction

Eukaryotic microalgae (excluding prokaryotic microalgae in this review) are classified into various phylogenetic divisions, including Chlorophyta (e.g., *Chlamydomonas reinhardtii* and *Chlorella vulgaris*), Rhodophyta (e.g., *Cyanidioschyzon merolae*), Heterokontophyta (e.g., the diatom *Phaeodactylum tricornutum*), Haptophyta (e.g., *Emiliania huxleyi*), Dinophyta (e.g., *Symbiodinium minutum*), and Euglenophyta (e.g., *Euglena gracilis*) [1]; their evolution, morphology, habitat, and metabolism are extremely diverse. Although the genomes of *C. reinhardtii* and *C. merolae* were sequenced prior to the genomes of other algal species [2,3], research using limited algal species is not sufficient to understand the biology of diverse microalgae. To gain this understanding, a wide range of algal species needs to be studied. Notably, as microalgae have photosynthetic ability, fast and autotrophic growth, and various material productivity, they have recently attracted attention because their biomass can be used to produce food, fuel, and other valuable materials, and outdoor culture equipment has been developed [4,5,6]. Under outdoor culture conditions, microalgae cannot avoid fluctuating environmental stresses, such as high light, low and high temperatures, and UV irradiation. Exposure to these environmental stresses increases the accumulation of reactive oxygen species (ROS), including H_2_O_2_, superoxide radical, hydroxyl radical, and singlet oxygen, as they are the byproducts of cellular oxygenic processes. The superoxide radical is generated by the reduction of molecular oxygen in the photosynthetic electron transport chain and the respiratory chain. It is then converted to H_2_O_2_ via the reaction with superoxide dismutase (SOD). The hydroxyl radical is generated from H_2_O_2_ in the Fenton reaction with free Cu^+^ or Fe^2+^. Singlet oxygen is generated by transferring the energy of the photoexcited pigments to molecular oxygen. At appropriate levels, ROS act as signaling molecules that regulate cellular activities, but when accumulated excessively, they oxidize nucleic acids, proteins, and lipids, leading to oxidative stress damage in cells [7,8]. To avoid ROS-induced cytotoxicity, organisms have developed various antioxidants, including carotenoids, ascorbate, and glutathione. These antioxidants are the key factors in determining the environmental stress tolerance and outdoor growth efficiency of microalgae [9]. This review describes recent findings regarding the diverse biosynthetic pathways and functions of these antioxidants which act to relieve environmental stress in microalgae.

## 2. Carotenoids

### 2.1. Carotenoid Compounds

**Figure 1:**
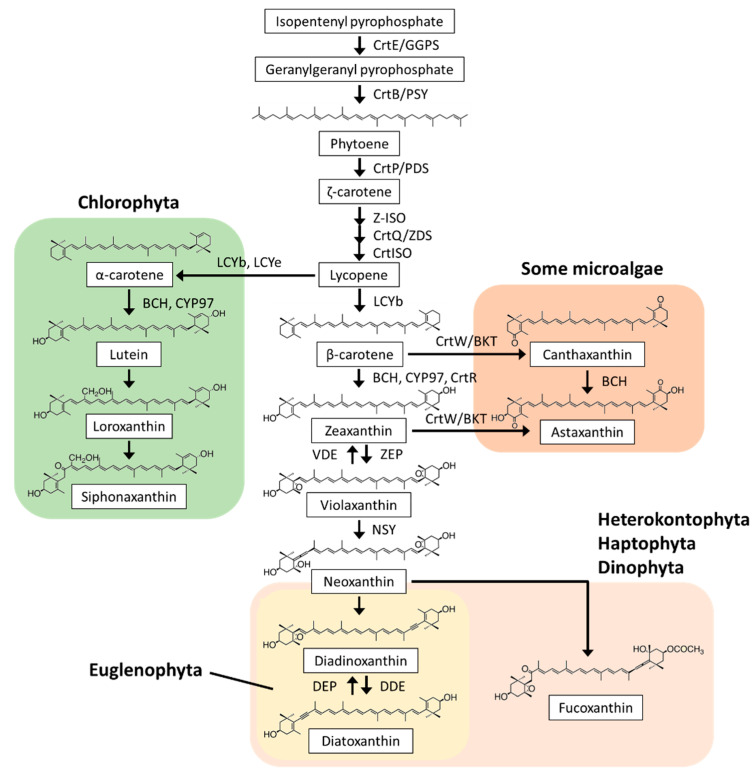
Carotenoid structures and biosynthesis in microalgae. Most microalgae contain a series of carotenoid compounds, from isopentenyl pyrophosphate to violaxanthin; the exception are red algae (e.g., *Cyanidioschyzon merolae*), which lack violaxanthin. α-carotene, lutein, loroxanthin, and siphonaxanthin are found in Chlorophyta, diadinoxanthin and diatoxathin are found in Heterokontophyta, Haptophyta, Dinophyta, and Euglenophyta, fucoxanthin is found in Heterokontophyta, Haptophyta, and Dinophyta, and canthaxanthin and astaxanthin are found in some microalgae (e.g., *Haematococcus pluvialis*, *Chromochloris zofingiensis*, and *Euglena gracilis*). The synthetic genes of loroxanthin, siphonaxanthin, diadinoxanthin, and fucoxanthin have not yet been identified. Violaxanthin and zeaxanthin are interconverted by VDE and ZEP, respectively, in the violaxanthin cycle via antheraxanthin; diadinoxanthin and diatoxanthin are similarly interconverted by DDE and DEP, respectively, in the diadinoxanthin cycle. CrtE/GGPS, geranylgeranyl pyrophosphate synthase; CrtB/PSY, phytoene synthase; CrtP, PDS, phytoene desaturase; Z-ISO, ζ-carotene isomerase; CrtQ/ZDS, ζ-carotene desaturase; CrtISO, prolycopene isomerase; LCYb, lycopene β-cyclase; LCYe, lycopene ε-cyclase; BCH, CYP97, and CrtR, carotene hydroxylase; ZEP, zeaxanthin epoxidase; VDE, violaxanthin de-epoxidase; NSY, neoxanthin synthase; DDE, diadinoxanthin de-epoxidase; DEP, diatoxanthin epoxidase; CrtW/BKT, β-carotene ketolase.

Carotenoids are isoprenoid compounds with C40 backbones, and their colors range from yellow to red. In nature, more than 750 carotenoid compounds have been structurally defined, and among them, at least 44 are found in eukaryotic microalgae. Most microalgae possess β-carotene and zeaxanthin, whereas other carotenoid compounds are extremely diverse depending on their phylogeny (Figure 1) [10,11]. Just like land plants, Chlorophyta species contain an abundance of β-carotene, lutein, neoxanthin, and violaxanthin. Specific carotenoid compounds, such as loroxanthin and siphonaxanthin, were also detected in Chlorophyta [10,11,12,13]. In macrophytic-type Rhodophyta (e.g., *Porphyra umbilicalis*), lutein is a major carotenoid compound, but it is absent in microphytic-type *C. merolae*, in which β-carotene and zeaxanthin are the predominant carotenoid compounds [14,15]. Diadinoxanthin and fucoxanthin are the major carotenoid compounds in Heterokontophyta, Haptophyta, and Dinophyta, whereas only diadinoxanthin is present in Euglenophyta [10,11,16,17,18,19]. In some microalgae (e.g., Chlorophyta species *Haematococcus pluvialis* and *Chromochloris zofingiensis*), astaxanthin synthesis is induced under various stress conditions, such as high light and high salinity [20]. Astaxanthin has also been detected in non-photosynthetic *E. gracilis*, suggesting that Euglenophyta can potentially synthesize this carotenoid compound [21]. In microalgae, these diverse carotenoid compounds are valuable chemotaxonomic biomarkers [10].

### 2.2. Carotenoid Biosynthesis

In eukaryotic microalgae, the biosynthetic pathway from phytoene to lycopene is conserved, whereas the downstream pathway from lycopene to each end carotenoid compound is diverse, as reviewed below (Figure 1).

#### 2.2.1. Lycopene Synthesis

Isopentenyl pyrophosphate (IPP), which is a C5 unit of the carotenoid backbone, is synthesized via the mevalonate (MVA) pathway or via the non-mevalonate (1-deoxy-D-xylulose 5-phosphate/2-C-methylerythritol 4-phosphate, DOXP/MEP) pathway. Most microalgae utilize the DOXP/MEP pathway, whereas the Euglenophyta species are exceptionally dependent on the MVA pathway [11,22]. IPP is added to farnesyl pyrophosphate (C15), produced from three IPP molecules, by geranylgeranyl pyrophosphate (GGPP, C20) synthase (CrtE, also called GGPS). Two GGPP molecules are condensed by phytoene synthase (CrtB, also called PSY) to produce phytoene (C40). Phytoene is the first carotenoid compound, and its synthesis is known to be the rate-limiting step in the carotenoid biosynthetic pathway [23]. It has been reported that mutations in the *PSY* gene in *C. reinhardtii* cause carotenoid deficiency, colorless cell appearance, and ROS accumulation [24,25], and *crtB* gene knockdown in *E. gracilis* showed a similar tendency [19].

Subsequently, phytoene is desaturated to 9,15,9′-tri-*cis*-ζ-carotene by phytoene desaturase (CrtP, also called PDS), isomerized to 9,9′-di-*cis* ζ-carotene by ζ-carotene isomerase (Z-ISO), desaturated to 7,9,7′,9′-tetra-*cis* lycopene (pro-lycopene) by ζ-carotene desaturase (CrtQ, also called ZDS), and isomerized to all-*trans* lycopene by prolycopene isomerase (CrtISO) [10,11]. *Cis*-carotenes are isomerized by Z-ISO and CrtISO in the dark and non-enzymatically photoisomerized in the light [26]. The genes encoding enzymes catalyzing six carotenoids synthesis steps from IPP to lycopene are widely conserved in microalgae.

#### 2.2.2. α-Carotene and Derivatives Synthesis

Lycopene is cyclized at both ends by lycopene cyclase (LCY). Distinct LCYb and LCYe enzymes generally form a β-ring at one end and an ε-ring at the other end of α-carotene, respectively [27]. A recent study reported that LCYb and LCYe from *Dunaliella bardawil* exhibit both β- and ε-cyclase activities [28]. *Ostreococcus lucimarinus* and its relatives have a unique gene encoding LCYb, LCYe, and a C-terminal light-harvesting complex (LHC, see Section 2.3.1) domain fusion protein in a single polypeptide [29]. α-Carotene is then hydroxylated to lutein by nonheme/di-iron carotene hydroxylase (BCH) and heme-containing cytochrome P450-type carotene hydroxylase (CYP97). BCH and CYP97A have hydroxylation activity towards the β-ring of α-carotene, and CYP97C have a hydroxylation activity towards the ε-ring of α-carotene [30,31]. The LCYb and CYP97 family enzymes are widely distributed in microalgae, whereas LCYe and CYP97C are involved in ε-ring formation and hydroxylation in Chlorophyta [32]. Therefore, the α-carotene, lutein, and downstream carotenoid compound synthetic pathways are specific to Chlorophyta. Loroxanthin, siphonaxanthin, prasinoxanthin, and monadoxanthin are considered to be synthesized from lutein, but the genes involved in their synthesis have not yet been identified.

#### 2.2.3. β-Carotene and Derivatives Synthesis

β-Carotene is produced by β-cyclization of lycopene at both ends. LCYb generally catalyzes this reaction [33], and *LCYb* genes are found in all microalgae. The β-rings of β-carotene are hydroxylated by BCH, CYP97, and CrtR (which is a third-type β-carotene hydroxylase homologous to BCH) to produce zeaxanthin. This step is highly diversified in carotenoid biosynthesis. Chlorophyta species have two types of β-carotene hydroxylases, BCH and CYP97A [31,34]. The microphytic red alga *C. merolae* possesses the *crtR* gene and lacks the *BCH* and *CYP97* genes [15]. In Heterokontophyta, Haptophyta, Dinophyta, and Euglenophyta, CYP97s (clans E, F, G, and H) are the sole β-carotene hydroxylases [32,35,36]. Lycopene β-cyclases and β-carotene hydroxylases have been demonstrated to be physiologically important for various environmental stress responses in microalgae. The halotolerant green alga *D. salina* upregulates the *LCYb* gene to accumulate β-carotene when exposed to saline, high light, and nitrogen depletion stresses [37]. In *P. tricornutum*, *CYP97* gene expression is induced in response to high light in order to accumulate β-carotene derivatives, fucoxanthin and diatoxanthin [35]. Our reverse genetic analysis revealed that *E. gracilis* CYP97H1 is essential for carotenoid synthesis and chloroplast homeostasis [36].

The resulting zeaxanthin is epoxidized to violaxanthin via antheraxanthin at both β-rings by zeaxanthin epoxidase (ZEP). In accordance with their carotenoid compositions, *ZEP* genes are found in Chlorophyta, Heterokontophyta, Haptophyta, Dinophyta, and Euglenophyta, but not in microphytic Rhodophyta [38]. *ZEP* genes in chlorophytes (*C. reinhardtii* and *C. zofingiensis*) and heterokontophytes (*P. tricornutum* and *Nannochloropsis oceanica*) have been shown to be functional [39,40,41,42]. Violaxanthin is then converted to neoxanthin through catalysis by neoxanthin synthase (NSY). The gene encoding NSY has been identified as an LCY paralog in tomatoes [43], but not yet in microalgae. Based on their chemical structures, specific carotenoid compounds, such as diadinoxanthin and fucoxanthin found in Heterokontophyta, Haptophyta, Dinophyta, and Euglenophyta, are predicted to be synthesized from neoxanthin, but their synthetic pathways have not yet been clarified [10,11].

Astaxanthin is produced by hydroxylation and ketolation of β-carotene at both β-rings. The ketolation reactions are catalyzed by β-carotene ketolase (CrtW/BKT) [10,11]. The *crtW* genes have been identified in Chlorophyta *H. pluvialis* and *C. zofingiensis* [44,45]. It has been reported that *C. zofingiensis* accumulates astaxanthin under high light, nitrogen deprivation, and high salinity conditions by upregulating *BCH* and *crtW* gene expression [46,47,48].

### 2.3. Carotenoid Functions

#### 2.3.1. Light Harvesting

Carotenoids bind to light-harvesting complexes (LHCs) with chlorophylls. Carotenoids in LHC promote photosynthesis by absorbing blue-green light and transferring energy to nearby chlorophylls [49]. The efficiency of this energy transfer varies depending on the carotenoid and chlorophyll compositions in the LHC. In addition to LHC, fucoxanthin-chlorophyll *a*/*c* binding proteins (FCP) in diatoms [50,51] and the peridinin-chlorophyll *a* protein complex (PCP) in dinoflagellates [52,53] also act as light-harvesting complexes binding specific carotenoids.

#### 2.3.2. Photoprotection

When photoexcited, chlorophyll transitions to the triplet state, after which it generates singlet oxygen by transferring energy from triplet chlorophyll to oxygen molecules. Singlet oxygen may damage the D1 subunit of photosystem II (PSII) and inhibit the repair of this subunit, leading to photoinhibition [54,55]. Carotenoids suppress singlet oxygen generation by receiving excess energy from triplet chlorophyll and dissipating this energy as heat. Carotenoids also directly receive energy from singlet oxygen and scavenge it [56]. In fact, *C. reinhardtii* mutant lacking carotenoid (the FN68 strain) is sensitive to light and unable to accumulate LHCs associated with both photosystems [57], demonstrating that quenching capacities of carotenoids against triplet chlorophyll and singlet oxygen contribute to photoprotection.

#### 2.3.3. Xanthophyll Cycles

Xanthophyll cycles control non-photochemical quenching (NPQ), which dissipates excessive light energy in the form of heat under high light conditions. There are two types of xanthophyll cycles: the violaxanthin cycle found in Chlorophyta and the diadinoxanthin cycle found in Heterokontophyta, Haptophyta, Dinophyta, and Euglenophyta. In the violaxanthin cycle, violaxanthin bound to the LHC of PSII is converted to zeaxanthin via antheraxanthin by violaxanthin de-epoxidase (VDE) under high light conditions to reduce the light harvesting efficiency (Figure 1). Under low light or dark conditions, zeaxanthin is converted back to violaxanthin via antheraxanthin by zeaxanthin epoxidase (ZEP) (Figure 1). Similarly, in the diadinoxanthin cycle, diadinoxanthin is converted to diatoxanthin by diadinoxanthin de-epoxidase (DDE) under high light conditions, and the reverse reaction is performed by diatoxanthin epoxidase (DEP) under low light or dark conditions. In Chlorophyta, *C. reinhardtii* VDE converts violaxanthin to zeaxanthin under high light conditions; however, it is not required for high light acclimation [39]. In contrast, *C. vulgaris* VDE-mediated zeaxanthin accumulation is crucial for the induction of NPQ under high light, suggesting diverse evolution of the violaxanthin cycle among Chlorophyta [58]. In diatoms, a silencing study suggested that *P. tricornutum* DDE, a VDE homolog, catalyzes diadinoxanthin de-epoxidation and induces NPQ under high light [59]. Moreover, it was reported that the culturing of *E. gracilis* at low temperatures results in photosensitivity and an increase in the ratio of diatoxanthin/diadinoxanthin, suggesting a functional diadinoxanthin cycle in Euglenophyta [60].

#### 2.3.4. Stabilization of Lipid Membranes

In microalgae, physical properties of lipid membranes are associated with cellular processes and environmental stress tolerance. Notably, the physical properties of thylakoid membranes affect the photosynthetic activity. Hydrophobic carotenoids are incorporated into lipid membranes, and xanthophylls containing polar groups at both ends are oriented across the lipid membranes. These carotenoids modify membrane fluidity and enhance its stability [61,62]. Physiological evidence of carotenoid-mediated membrane stability has been documented in violaxanthin de-epoxidation in plants [63]. A recent study reported that diadinoxanthin de-epoxidation in the thylakoid membrane of *P. tricornutum* causes membrane rearrangement and confers stabilization and rigidification to membranes [64]. 

#### 2.3.5. Eyespot Formation for Phototaxis

Carotenoids are also major components of eyespot globules found in flagellated microalgae and are essential for phototactic responses. Phototaxis is a responsive movement in which the swimming direction changes to optimize photosynthetic activity depending on the light intensity [65,66]. In *C. reinhardtii*, an eyespot is formed in the chloroplasts, and two carotenoid-rich layers reflect light from outside the cell and amplify the light signal received by the photoreceptor, or they shade light from inside the cell to accurately recognize the light direction [67]. The eyespot of *E. gracilis* is positioned in the cytosol near the base of the major flagellum, its development is independent of chloroplast development, and it has been demonstrated that its presence is required for initiating phototaxis [21,68]. These findings suggest that eyespot position and physiological function differ between Chlorophyta and Euglenophyta.

## 3. Ascorbate

The hydrophilic antioxidant ascorbate (AsA) accumulates at high (millimolar) concentrations in cells and plays a crucial role in photooxidative stress defense in all microalgae.

### 3.1. Ascorbate Biosynthesis

Photosynthetic organisms and most animals, except for humans and some others, can synthesize AsA. In photosynthetic organisms, AsA biosynthetic pathways are classified into the plant pathway (also called the D-mannose/L-galactose pathway) and the *Euglena* pathway (also called the D-galacturonate pathway).

#### 3.1.1. Plant Pathway

**Figure 2:**
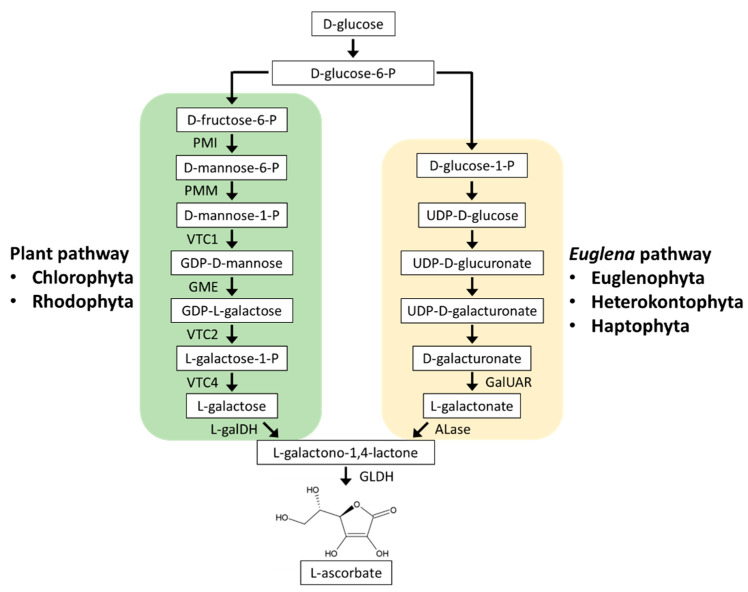
Ascorbate biosynthesis in microalgae. Microalgae use two distinct pathways: plant pathway (D-mannose/L-galactose pathway) or *Euglena* pathway (D-galacturonate pathway). Chlorophyta and Euglenophyta have been demonstrated to use the plant pathway and *Euglena* pathway, respectively. Rhodophyta lacking the *VTC2* homolog are predicted to use a modified plant pathway including alternative L-galactose phosphorylase instead of VTC2. Heterokontophyta and Haptophyta are predicted to use the *Euglena* pathway. PMI, phosphomannose isomerase; PMM, phosphomannomutase; VTC1, GDP-L-mannose pyrophosphorylase; GME, GDP-D-mannose-3’,5’-epimerase; VTC2, L-galactose phosphorylase; VTC4, L-galactose-1-P phosphatase; L-galDH, L-galactose dehydrogenase; GLDH, L-galactono-1,4-lactone dehydrogenase; GalUAR, D-galacturonic acid reductase; ALase, aldonolactonase.

In the plant pathway (Figure 2), D-glucose-6-phosphate (P) is stepwise converted to GDP-L-galactose via D-fructose-6-P, D-mannose-6-P, D-mannose-1-P, and GDP-D-mannose. GDP-L-galactose is then converted to L-galactose by GDP-L-galactose phosphorylase (VTC2) and L-galactose-1-P phosphatase (VTC4). L-galactose is dehydrogenated to L-galactono-1,4-lactone by L-galactose dehydrogenase (L-galDH) and finally to AsA by L-galactono-1,4-lactone dehydrogenase (GLDH) [69,70]. AsA is distributed in most cellular compartments; however, the final AsA synthesis step by GLDH occurs in the mitochondria, and the others occur in the cytosol [71,72]. Therefore, the synthesized AsA is transported from the mitochondria to the chloroplasts, where AsA is the most abundant, and to other compartments [73]. 

All of these plant pathway genes have been identified in *A. thaliana* and have been reported to be conserved in Chlorophyta *C. reinhardtii*, *V. carteri*, *Chlorella* sp. NC64A, and *Coccomyxa* sp. C169 [74]. The enzymatic property of *C. reinhardtii* VTC2, a key enzyme of the plant pathway, was found to be similar to those of *A. thaliana* VTC2, and its knockdown resulted in a 90% decrease in AsA content. Moreover, in *C. reinhardtii*, the transition from dark to light, high light irradiation, and H_2_O_2_ treatment caused *VTC2* gene upregulation and AsA accumulation [74,75]. These findings suggested that AsA synthesis via the plant pathway protects *C. reinhardtii* cells from photooxidative stress. 

In contrast to land plants and Chlorophyta, Rhodophyta lack the *VTC2* homologous gene. However, supplementation experiments of plant pathway intermediates and positional isotopic labeling approach suggested that Rhodophyta synthesized AsA via a plant-like pathway. Therefore, Rhodophyta may use a modified plant pathway by the catalysis of an unidentified enzyme that converts GDP-L-galactose to L-galactose instead of VTC2 [70].

#### 3.1.2. Euglena Pathway

The *Euglena* pathway was proposed after the detection of D-galacturonate and L-galactono-1,4-lactone as AsA biosynthesis intermediates in *E. gracilis* (Figure 2) [76]. This pathway was then supported by genetic and biochemical characterizations of D-galacturonic acid reductase (GalUAR) and aldonolactonase (ALase) in *E. gracilis* [77,78]. GalUAR reduces D-galacturonate to L-galactonate, which is then converted to L-galactono-1,4-lactone by ALase. The final step that converts L-galactono-1,4-lactone to AsA by GLDH is common in both plant and *Euglena* pathways. The fact that growth inhibition of ALase-knockdown *E. gracilis* can be counteracted by supplementation with L-galactono-1,4-lactone indicated that in *E. gracilis*, the *Euglena* pathway is predominantly utilized for AsA biosynthesis [78]. The *Euglena* pathway-specific *ALase* gene is homologous to that in the diatoms *P. tricornutum* and *Thalassiosira pseudonana*, but not to that in *A. thaliana*, *C. reinhardtii*, and *V. carteri*, suggesting the utilization of this pathway in diatoms [78]. Genome sequencing and phylogenetic analyses predicted that Heterokontophyta other than diatoms, Haptophyta, and Cryptophyta also use the *Euglena* pathway [70,79,80].

In *E. gracilis*, light irradiation induces ALase activity and AsA accumulation [78]. The photoinduction of AsA in this algal species is specific to blue light, but not to red and green light [81]. In the diatom *Skeletonema marinoi*, strong blue light irradiation induces AsA synthesis along with the synthesis of photosynthetic pigments [82]. Therefore, AsA biosynthesis is considered to be sensitive to the light environment in a wide range of microalgae, regardless of whether they drive either plant or *Euglena* pathways.

### 3.2. Ascorbate Functions

#### 3.2.1. Ascorbate Peroxidase and the Ascorbate-regenerating System

**Figure 3:**
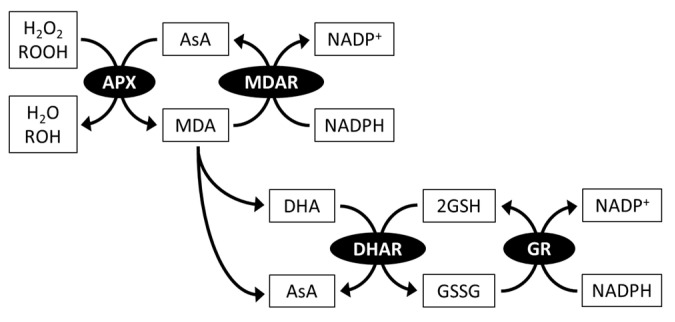
Ascorbate (AsA)-glutathione (GSH) cycle. All or some of genes and activities of AsA-GSH components have been identified in all microalgal divisions. APX, ascorbate peroxidase; ROOH, organic hydroperoxides; MDA, monodehydroascorbate; MDAR, MDA reductase; DHA, dehydroascorbate; DHAR, DHA reductase; GSSG, oxidized glutathione; GR, glutathione reductase.

AsA is an electron donor of the ROS-scavenging enzyme ascorbate peroxidase (APX) which catalyzes the reduction of H_2_O_2_ to H_2_O and prevents oxidative stress damage in cells (Figure 3) [83,84]. The rate constant of APX for scavenging H_2_O_2_ (10^7^) is much higher than that of AsA itself (up to 6); thus, APX activity allows rapid avoidance of H_2_O_2_ toxicity [8]. APX also has a reduction activity towards organic hydroperoxides, but this activity is lower than that of H_2_O_2_ [83,85], suggesting that APX is an enzyme specialized for H_2_O_2_ scavenging.

During the APX reaction, APX simultaneously produces monodehydroascorbate (MDA), which is a univalent oxidant of AsA. MDA is then spontaneously disproportionated to AsA and dehydroascorbate (DHA), a divalent oxidant of AsA. MDA and DHA are reduced back to AsA by MDA reductase (MDAR) using NADPH as an electron donor, and DHA reductase (DHAR) using glutathione (GSH) as an electron donor, respectively. The resulting oxidized form of glutathione (GSSG) is reduced back to GSH by glutathione reductase (GR) using NADPH as an electron donor. This AsA-regenerating system is termed the AsA-GSH cycle and is essential for maintaining AsA redox homeostasis (Figure 3) [84,86].

The number and localization (including predictions) of some microalgae AsA-GSH cycle enzymes have been documented. *C. reinhardtii* contains three APX isoforms; APX1 and APX2 were predicted to be dual-targeted in chloroplasts and mitochondria, and APX4 in chloroplasts [87]. Single MDAR and DHAR enzymes are present in *C. reinhardtii*, and they are probably located in the cytosol [88,89]. *C. reinhardtii* GRs are composed of two isoforms [90]. *E. gracilis* contains APX, MDAR, DHAR, and GR enzymes as a single isoform, all of which are localized in the cytosol [91,92,93]. Therefore, AsA regeneration is functional only in the cytosol, at least in *C. reinhardtii* and *E. gracilis*. In other microalgal species, two APX isoforms in the cytosol and chloroplasts of *C. merolae* and four APX isoforms in the cytosol and peroxisomes of *Galdieria sulphuraria* have been reported [94]. In contrast to microalgae, *A. thaliana* contains more AsA-GSH cycle enzyme sets, which are composed of eight APX, five MDAR, three DHAR, and two GR isoforms and are widely distributed in the cytosol, chloroplasts, mitochondria, and peroxisomes [95]. Microalgae that live in water environments are less exposed to oxygen and light, which stimulates ROS generation than that in land plants. Thus, it can be presumed that in microalgae, the number and localization of AsA-GSH cycle enzymes were more limited than those in land plants during evolution.

A recent study reported that in *C. reinhardtii*, the expression of *APX* genes is induced under high light stress, and a knockdown of chloroplastic *APX4* caused sensitivity to photo-oxidative stress [87]. Moreover, overexpression and knockdown of *MDAR* and *DHAR* genes in *C. reinhardtii* resulted in tolerance and sensitivity to high light stress, respectively [88,89]. In *E. gracilis*, APX-knockdown cells showed high H_2_O_2_ accumulation [91]. These findings demonstrated that the microalgal AsA-GSH cycle plays a key role in photooxidative stress defense.

#### 3.2.2. Reductant for Xanthophyll Cycles

Furthermore, AsA is used as a reductant of VDE and DDE reactions in xanthophyll cycles and is thus required for maintaining appropriate NPQ levels in photosynthetic organisms (see Section 2.3.3.) [96]. It has been reported that in *C. vulgaris* and *P. tricornutum*, VDE enzymes are active in the presence of AsA in vitro [58,97]. However, a recent study using *C. reinhardtii* demonstrated that AsA deficiency caused by *vtc2* knockout does not limit violaxanthin de-epoxidation and NPQ induction [98]. Therefore, the role of AsA as a reductant in the xanthophyll cycle of microalgae remains controversial.

## 4. Glutathione

GSH is a low molecular weight thiol tripeptide found in all organisms. It is composed of Glu, Cys, and Gly, plays an important role as a hydrophilic antioxidant and thiol-based redox regulator, and is essential for the survival of microalgae. It is also used for the biosynthesis of phytochelatins and trypanothione (GSH derivatives).

### 4.1. Glutathione Biosynthesis

**Figure 4:**
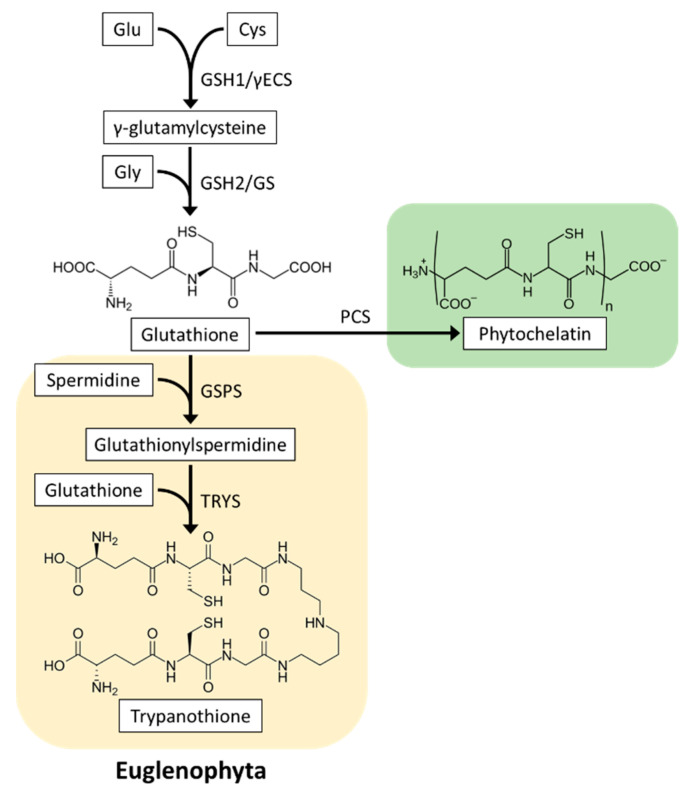
Structure and biosynthesis of glutathione, phytochelatins, and trypanothione in microalgae. Glutathione biosynthesis is conserved in all biological kingdoms. Phytochelatin biosynthesis occurs in most microalgae, whereas among photosynthetic organisms, trypanothione is detected only in Euglenophyta. GSH1/γECS, γ-glutamylcysteine synthetase; GSH2/GS, glutathione synthetase; PCS, phytochelatin synthase; GSPS, glutathionylspermidine synthetase; TRYS, trypanothione synthetase.

GSH is synthesized in two ATP-dependent steps catalyzed by γ-glutamylcysteine synthetase (GSH1, also abbreviated as γECS) and glutathione synthetase (GSH2, also abbreviated as GS). In the first step, GSH1 ligates Cys with Glu to produce γEC. In the second step, Gly is ligated to γ-EC by GSH2 to yield GSH [99] (Figure 4). Two GSH biosynthesis genes are conserved in all biological kingdoms. Genetic and physiological analyses using *A. thaliana* mutants have demonstrated that both GSH1 and GSH2 are essential for the development of plant roots and seedlings [100,101]. One study reported that glutathione synthesis in *E. gracilis* grown in the dark was photoinduced post-transcriptionally [102]. In Chlorophyta, glutathione synthesis is downregulated by cold and superoxide generator treatment in *D. viridis* [103] and by high light in *C. reinhardtii* [89]. These findings suggested that microalgae acclimate to environmental stresses by altering cellular glutathione levels. However, to our knowledge, the glutathione synthetic genes in microalgae have not yet been characterized, and thus, the physiological significance of glutathione synthesis is poorly understood.

### 4.2. Glutathione Functions

#### 4.2.1. Glutathione Peroxidase

**Figure 5:**
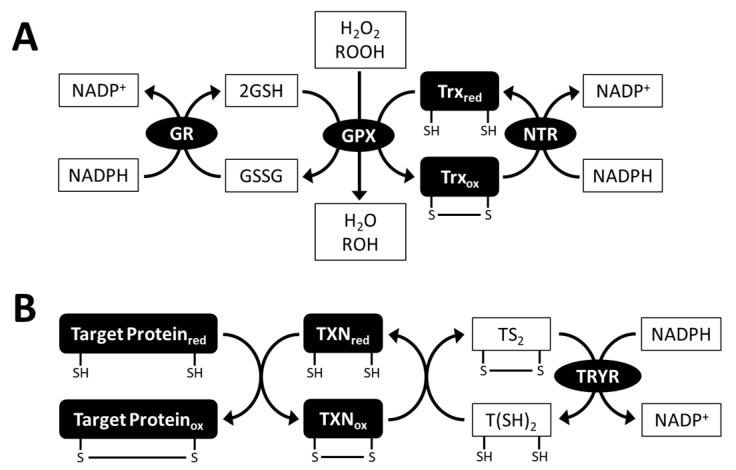
Glutathione peroxidase (GPX) reaction (**A**) and trypanothione (T(SH)_2_) system (**B**). GPX uses glutathione (GSH) or thioredoxin (Trx) as electron donors. T(SH)_2_ system is biochemically and physiologically characterized in trypanosomatids, and *Euglena gracilis* contains a set of genes encoding the components of the T(SH)_2_ system. GSSG, oxidized glutathione; GR, glutathione reductase; NTR, NADPH-dependent thioredoxin reductase; TS_2_, oxidized trypanothione; TRYR, trypanothione reductase; TXN, tryparedoxin; red, reduced form; ox, oxidized form.

Glutathione peroxidase (GPX) is an antioxidant enzyme that reduces H_2_O_2_, organic hydroperoxides and lipid peroxides, and detoxifies them using GSH or thioredoxin (Trx) as electron donors. During the GPX reaction, GSSG and oxidized Trx are reduced by GR and NADPH-dependent Trx reductase (NTR) (Figure 5A). GPX is classified into two types: enzyme-containing selenocysteine (SeCys) at the catalytic site and enzyme without SeCys [104,105]. *C. reinhardtii* contains five genes encoding GPXs, including both SeCys-containing (GPX1 and GPX2) and non-selenium GPXs (GPX3, GPX4, and GPX5). These GPX enzymes are predicted to be distributed in cellular compartments, including the cytosol, chloroplasts, and mitochondria [106,107]. To date, their functional characterization has been focused on *C. reinhardtii* GPX5, which uses Trx as an electron donor, and its gene expression is responsive to high light and singlet oxygen generators [108]. Knockout of *GPX5* in *C. reinhardtii* causes ROS accumulation and thereby arrests growth, suggesting the crucial role of GPX5 as an antioxidant enzyme [109]. However, little is known about the physiological functions of GSH-dependent GPX in *C. reinhardtii*. *Chlorella* sp. NJ-18 contains two genes encoding non-selenium GPXs, which use Trx as an electron donor. These *GPX* genes are upregulated in response to singlet oxygen generator treatment and UV-B irradiation [110]. Unlike non-selenium GPXs from Chlorophyta, non-selenium GPX isolated from *E. gracilis* uses GSH as an electron donor [111]. In addition to GSH-dependent GPX, the transcriptome data of *E. gracilis* indicated the existence of three putative Trx-dependent GPXs [112].

#### 4.2.2. Ascorbate Regeneration

As described in Section 3.1.1. GSH is involved in AsA regeneration by providing an electron donor for DHAR. In addition, GSH itself contributes to the non-enzymatic reduction of DHA to AsA in high pH environments. A recent study using *A. thaliana* demonstrated that DHAR activity and GSH content cooperatively act as DHA reductants under high light stress conditions [113]. Non-enzymatic DHA regeneration by GSH is assumed to be functional in microalgae.

#### 4.2.3. Heavy Metal Detoxification

Exposure of microalgae to heavy metal ions, such as cadmium, copper, and zinc, causes ROS production and cytotoxicity. GSH and phytochelatins (PCs), which are GSH polymers found in most microalgae, bind to heavy metal ions and detoxify them [114,115]. The general formula of PCs is represented as (γGlu-Cys)_n_-Gly (PC_n_), and microalgae can synthesize those ranging from PC_2_ to PC_6_ [116,117,118,119,120]. PC synthesis is catalyzed by phytochelatin synthase (PCS), which binds two molecules of GSH to produce PC_2_ or GSH and PC_n_ to PC_n+1_ (Figure 4); therefore, cellular levels of GSH and its precursor γEC are the key factors in PC synthesis induction. In addition, PCS is activated in the presence of heavy metal ions and promotes PC synthesis [121]. It has been reported that a wide range of microalgae (Chlorophyta *C. reinhardtii* and *D. tertiolecta*, Rhodophyta *C. merolae*, diatoms *P. tricornutum* and *Thalassiosira weissflogii*, and Euglenophyta *E. gracilis*) markedly induced γEC, GSH, and PC synthesis and resisted heavy metal toxicity when exposed to cadmium [116,117,118,119,120,122,123,124,125]. Moreover, the heterologous expression of *PCS* genes from *C. merolae* and *E. gracilis* in yeast confers Cd^2+^ tolerance [118,126]. These findings explain the physiological importance of GSH and PC accumulation in microalgae for heavy metal detoxification.

#### 4.2.4. Glutathione Derivative Trypanothione

Tyrpanothione (*N*^1^,*N*^8^-bis(glutathionyl)spermidine, T(SH)_2_) is a specific thiol-based antioxidant found in Euglenophyta and phylogenetically related trypanosomatid parasites [127]. Its biosynthesis is catalyzed by two distinct enzymes: glutathionylspermidine (GSP) synthetase (GSPS) conjugates the first GSH molecule to spermidine, and trypanothione synthetase (TRYS) adds the second GSH molecule to GSP (Figure 4) [128]. Transcriptome data showed that *E. gracilis* contains two highly homologous genes to GSPS and TRYS genes from trypanosomatid *Crithidia fasciculata* [112]; however, these genes have not yet been functionally characterized.

In trypanosomatids, the T(SH)_2_ system, which consists of T(SH)_2_, trypanothione reductase (TRYR), and Trx family protein tryparedoxin (TXN), activates target proteins via a dithiol/disulfide exchange reaction (Figure 5B) [128]. Target proteins of the trypanothione system include peroxiredoxin, which is a thiol peroxidase involved in oxidative stress defense. In addition, T(SH)_2_ is able to reduce DHA, thus contributing to APX-dependent ROS scavenging [129]. It has been demonstrated that the T(SH)_2_ system plays a crucial role in the survival of parasites exposed to oxidative stress in the host [130,131]. In *E. gracilis*, the TRYR enzyme was purified from algal cells and biochemically characterized [132]. Genes encoding putative TRYR and TXN were identified in the *E. gracilis* transcriptome data [112]. Moreover, knockdown of *TRYR* genes in *E. gracilis* inhibited growth, suggesting a functional T(SH)_2_ system in this algal species [133].

#### 4.2.5. Glutathione-Mediated Redox Regulations

GSH is also known to be involved in redox regulation of photosynthesis and the cell cycle. A previous study using *C. reinhardtii* identified 10 Calvin cycle enzymes that underwent protein S-glutathionylation, which is a post-translational modification in which GSH is added to the Cys residue of protein under oxidative stress conditions. Among them, the activities of phosphoribulokinase (PRK) and glyceraldehyde-3-phosphate dehydrogenase (GAPDH) were demonstrated to be modified by S-glutathionylation [134]. 

Cell cycle progression is regulated by nuclear GSH. It has been reported that the cell cycle was arrested at the G_1_ checkpoint in tobacco cell suspension cultures depleted of GSH levels [100,135]. However, these GSH-mediated redox regulations in microalgae are not fully understood at present, and thus, further investigation is necessary.

## 5. Conclusion and Future Perspectives

In all organisms, antioxidant biosynthesis and functions are key factors that determine environmental stress tolerance and cellular process maintenance. Microalgae have evolved antioxidant biosynthesis and function depending on their phylogenetic diversity. As a result, specific carotenoid compounds, such as diadinoxanthin, fucoxanthin, and astaxanthin, as well as the glutathione derivative trypanothione, and many distinct biosynthetic pathways occur in microalgae. This is essential for understanding the cellular metabolism and evolutionary processes of microalgae; however, the findings obtained to date may not be sufficient. Recently, the application of transgenic and genome editing technologies to study microalgae has enabled the modification of their metabolism. Modifications of antioxidant biosynthesis encouraged microalgae researchers to produce high levels of antioxidants and confer resistance to environmental stress. Importantly, as specific carotenoids, such as astaxanthin and fucoxanthin, are known to be effective in maintaining health and preventing disease in humans, carotenoid biosynthesis in microalgae has been actively studied as an attractive target for metabolic modification. In order to understand the evolution and physiology of antioxidants in microalgae and to be able to flexibly design them, future studies should further elucidate their pathways, regulatory mechanisms, and functions.

## Data Availability

The data presented in this study are available in this article.
